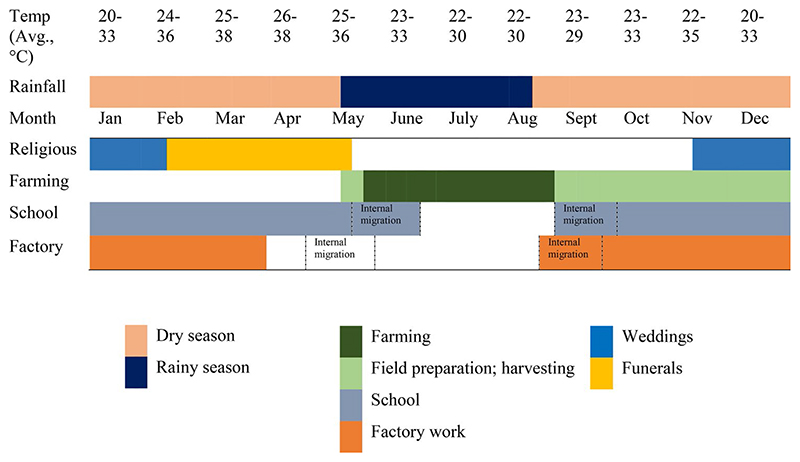# The issue is not ‘compliance’: exploring exposure to malaria vector bites through social dynamics in Burkina Faso

**DOI:** 10.1080/13648470.2021.1884185

**Published:** 2021-05-10

**Authors:** Federica Guglielmo, Hilary Ranson, N’falé Sagnon, Caroline Jones

**Affiliations:** a Vector Biology Department, Liverpool School of Tropical Medicine, Liverpool, UK; b Centre National de Recherche et Formation sur le Paludisme, Ouagadougou, Burkina Faso; c KEMRI-Wellcome Trust Research Programme, Kilifi, Kenya; d Centre for Tropical Medicine and Global Health, Nuffield Department of Medicine, University of Oxford, Oxford, UK

**Keywords:** Bednets, compliance, lifeways, exposure, ethnography, assemblage

## Abstract

Credited with averting almost 68% of new cases between 2000 and 2015, insecticide-treated bednets (ITNs) are one of the most efficacious malaria-prevention tools. Their effectiveness, however, depends on if and how they are used, making ‘compliance’ (and the social factors affecting it) a key area of interest for research on malaria transmission. This article situates the notion of compliance with ‘bednet use’ within everyday practices in an area of south-west Burkina Faso with high malaria transmission. By drawing on ethnographic fieldwork conducted between 2017 and 2018, it critically describes the precarious micro-environments that foreground bednet use—from gender and age to the means of (re)production of social and labour conditions—and assesses the bednets’ effectiveness and community uptake. Bednet use stems from concrete, ordinary dynamics that interweave only apparently at the margins of the time individuals most need to be protected by a net. This work conceptualises ‘compliance’ beyond binary indicators of intervention uptake and locates ‘use’ as the result of contingent assemblages.

## Introduction

By the end of 2015, more than one billion bednets had been distributed to populations living in malaria-endemic countries, resulting in an estimated 446 million infections averted in Sub-Saharan Africa—a region bearing more than 90% of the global malaria burden (AMP 2020; Bhatt, et al. 2015). However, by 2017, this phenomenal progress towards malaria elimination had stalled and begun to show signs of reversion in some countries (WHO 2018). Uneven intervention coverage between and within countries, lack of funding, and increasing resistance of malaria vectors (*Anopheles* mosquitoes) and parasites *(Plasmodia)* to insecticides and treatment were identified among the main explanations for this shift (WHO 2018). In response, a group of global health researchers called for additional funding to widen interventions—including bednet delivery—and greater accountability on the part of national governments in malaria-endemic countries, renewing their call to eradicate malaria within a generation (Feachem et al. 2019; for a critical appraisal, cf. WHO 2019a; Packard 2007). This proposition relies on a technology-centred ideology. Prioritising international biomedical expertise over the importance of context, and confiding in technology to overcome any constraints posed by social circumstances (including infrastructures, labour conditions, housing structures, etc.), the argument echoes previous eradication campaigns (Chandler and Beisel 2017; Cueto 2013). Widening bednet delivery would improve access to the tool; nevertheless, the efficacy of bednets as a public health intervention foregrounds the issue of ‘compliance’, highlighting public health assumptions on the seamless conformity of the beneficiaries with the behaviours required for an effective intervention.

Taking bednets for malaria control as an example, this paper interrogates this notion of compliance and re-frames it in reference to the structural circumstances in which the beneficiaries’ behaviour surfaces as ‘non/compliant’ Drawing on ethnographic fieldwork conducted in three rural communities in Burkina Faso, we explore the praxis of compliance from the perspective of bednet users within their specific lifeways. Intrinsically depending on an external structure, as compliance ‘with’ an intervention, we argue that thinking of ‘compliance as assemblage’ (cf. Brodwin 2010) helps us decentre the concept—a theoretical shift especially relevant in the case of devices or treatment designed to become an integral part of everyday life.

### Bednets: use and its assessment

Malaria is transmitted by female *Anopheles* mosquitoes infected with the *Plasmodium* parasite; nevertheless, the human-vector-parasite entanglement from which it surfaces extends beyond its immediate exchange and is inscribed within complex ‘material, semiotic, and biotic realities’ (Chandler and Beisel 2017, 414). Infrastructures may provide both fertile ground for mosquito breeding sites and limited access to health treatment, turning the infected body into a reservoir for the parasite. Micro-ecologies further determine mosquito species composition (Godfray 2013), susceptibility to insecticides (Ranson, et al. 2011), and shifting feeding and resting behaviour (Sougoufara, et al. 2017). Insecticide-treated nets (ITNs), target the entangled nature of these multiple dimensions.

ITNs insulate the human against other species, such as viruses, parasites, and their vectors (Nading 2014, 9). The insecticides target pregnant *Anopheles*, aiming to reduce the vector population; the physical barrier orders (by separating, merging, or both) spaces, bodies, species, and practices. In endemic countries, the ‘correct’ use of bednets entails sleeping under a net during Anophelines’ feeding hours (18:00–06:00) throughout the year; bednets are designed to be shared by co-sleepers (regardless of gender and age) and for indoor use. While bednets are thought of as a life-saving, minimally intrusive technology (Lindsay and Gibson 1988), the inadequacy of scalability in addressing the mosquito-parasite-human entanglement surfaces at the intersection between everyday practices of malaria control and the vertical nature of global interventions (Kelly and Beisel 2011). Anthropological research on these entanglements (Beisel 2015; Nading 2014) includes institutional actors (Eckl 2014), local actors and diagnostic device uptake (Chandler, et al. 2011), as well as data production: Marleen Tichenor (2017) explored the role of standardised measurements in shaping relationships between malaria actors and stakeholders, reified into numerical entities for the consumption of funding agencies. Measures of bednet use and approaches to determining compliance are a good example of this reification. To assess bednet use, standardised surveys ask whether the interviewee and any family members ‘slept under a bednet’ the preceding night (USAID 2016, Q.128–129), crystallising individual conformity with the clinical prescription into binary indicators, and imagining compliance as a coherent and stable response. This approach is oblivious of how long respondents were actually under a bednet, omitting crucial information regarding the relationship between bednet use and material protection. However, except for Dunn, Le Mare and Makungu’s (2016) study of cultural topologies related to funerals in Tanzania, relatively little attention has been devoted to theorising ‘compliance’ with bednet use beyond an intrinsically post-positivist paradigm, reiterating and prioritising biomedicine over the lifeways of the beneficiaries.

### Assemblages of compliance

‘Compliance’ entails a relationship between a knowledgeable provider and a passive receiver, becoming—within the ordering structure of the biomedical encounter—terrain for ideological affirmation (Trostle 1988). Eliciting forms of both control and rebellion, in which non-compliance surfaces as an assertion of autonomy and resistance, personal politics and self-determination (Van Dongen in Reynolds Whyte, et al. 2002, 71), compliance translates into a moral matter. The bare availability of a ‘life-saving’ device, technologically mediated in the case of bednets, turns compliance into a self-evident good (Applbaum and Oldani 2010) and its ‘proper’ enactment into a moral choice (Verbeek 2011). Global interventions on malaria reflect this dynamic, in so doing applying the neo-liberal blame of individual volitions to deterministic cultural and psychological predispositions, targeted through a biomedical lens (Whitmarsh 2013).

Colonial tropes about the beneficiaries’ ignorance and carelessness (Biruk and Prince 2008, 237; Chandler and Beisel 2017, 413) reverberate through similar strategies and in distorted representations of community behaviour. This is the case of Direct Observations of Treatment (DOT) for tuberculosis in India (Harper 2010); of Nigerian mothers from pastoralist groups blamed for delaying seeking treatment for their children (Kwabe et al. 2013); and, throughout the continent, of the strongly criticised repurposing of bednets for fishing, despite being based only on anecdotal evidence (Short et al. 2018). The Presidential Malaria Initiative (PMI), a leading funding body supporting bednet procurement in Burkina Faso, explained some bednet distribution shortcomings in 2016 through ‘inflated pre-campaign census […] since households often want to take advantage of additional free nets’ (PMI 2019, 23–24). These considerations share a framework based on blame, disregarding the shifting micro-politics, micro-practices, and micro-ecologies that converge in the efficacy of bednet use.

To account for the micro-dimensions co-existing within the performance of compliance, we adopt the notion of ‘assemblage’—an ‘anti-structural concept’ (Marcus and Saka 2006, 101) that allows the researcher to speak of emerging and decentred phenomena without omitting the structure of social life. By designating causes and processes as ‘immanent in an open system of intensities’ under an external force, assemblages result from the intersections of open systems (Marcus and Saka 2006, 103). Closely related to actor-network theory, the concept of assemblage allows us to think about why and how orders that involve actors different in nature—individuals, objects, and the relationships and ideologies that connect them—emerge in particular ways (Müller 2015, 27), expanding our understanding of the structure-agency dialectic. Paul Brodwin (2010) highlights this by examining the production and use of the ‘medication cassette’ in psychiatric care, where the notion of assemblage underlines the shifting and heterogeneous elements that enable compliance; these elements can function outside the assemblage they actualise, as well as in temporary but meaningful realities. We adopt this definition outside the enclosed clinical trajectory to frame ordinary modes of being that constitute the uneven, ever-shifting ontologies of bednet use. We show that low ‘compliance’ rates stem from the unidimensional and unidirectional nature of the questions asked providing an assessment loyal to the hierarchical relationship between providers and beneficiaries but revealing only a fractional aspect of how compliance is performed—i.e. from applying an inadequate external force.

## Methods

This study was part of ‘Improving the efficacy of malaria prevention in an insecticide-resistant Africa (MIRA)’, an interdisciplinary project investigating the reasons behind the persistence of malaria in the Comoé region in south-west Burkina Faso. Between 2017 and 2018, the lead author conducted 14 months of ethnographic fieldwork in three communities—Toma, Niakore, and Ouloumeni, hosting between 1500 and 3000 inhabitants (Crawl 2010)—which we selected on the grounds of their different ethnic backgrounds and of their proximity to the town of Banfora.

The main methods of data collection were participant observation, conversational interviews (individual and group), and semi-structured interviews. In each community, the primary participants consisted of a cluster of households (22 in Toma, 16 in Niakore, and nine in Ouloumeni), whose heads agreed to host the ethnographer for the study. Environmental factors influenced the numbers of recruited compounds: compounds were separated by interspersed fields in Niakore and Ouloumeni but were closer together in Toma, where the government confiscated the arable land in the 1970s. Data collection involved the extended networks of these households, including temporary guests, family members, and friends within the community. This approach resulted in a total of 46 semi-structured interviews with caregivers (mostly women) and household heads (mostly men). An interpreter from each community provided support in Fulfulde, Toussian, Karaboro and Turka. The data presented here primarily derive from participant observation and everyday interactions, conducted in French and occasionally in Jula, the vernacular language of the area. The non-English terms reported in this article are all in Jula.

We sought the consent of all participants for data collection (including frequent notetaking and the use of a digital audio recording device during informal interactions) and publication. For participant observation, conversational interviews and informal focus groups, consent was oral and iterative; otherwise, we required written consent, acquired with the support of a witness chosen by the participant if the participant was illiterate.

Ethical approval was obtained from the Liverpool School of Tropical Medicine (UK) (16-047), the Comité Ethique de Recherche en Santé of the Ministry of Health (2016-10-119), and the Comité Institutionel de Bioethique of the Centre National de Recherche et Formation sur le Paludisme (BF) (2016/006/MS/SG/CNRFP/CIB). To protect the confidentiality of the research participants, we changed the community toponyms and pseudo-anonymised direct quotations.

## Background

Burkina Faso is one of the countries hardest-hit by malaria, with an estimated 8 million cases in 2018 alone (WHO 2019b). Since the introduction of national bednet distribution campaigns in 2010, the country has witnessed improvements in bednet ownership and use (Samadoulougou et al. 2017). The indicators are still below the 80% coverage that would promote a ‘community-wide effect’ (Koenker 2018); modest increases will remain insufficient given the high level of insecticide-resistance displayed by local mosquito populations (Soma et al. 2020).

The National Malaria Control Programme (NMCP) organises bednet distributions every three years. Following a nation-wide census, households receive one bednet for every two individuals, with a cap of three bednets per household; in polygynous households, the cap applies to each wife rather than the husband (i.e. a household composed of one husband and three wives will receive up to nine bednets instead of three). However, bednets rarely survive the full three years, and the capped distribution results in the mere process of bednet replacement, undermining the universal coverage goal. At the time of this study, the latest distribution had occurred between seven and 20 months prior, at the end of the rainy season of 2016.

The Comoé region is holoendemic for malaria; peak-transmission falls during and right after the rainy season, from mid-May until November. A dry and progressively colder season replaces the rains until January, followed by increasing heat in March (*funtenikalo*, lit. ‘the month of heat’), April (*funtenibakalo*, ‘the month of great heat’), and May (*samiɲadonkalo*, ‘the beginning of winter’). Shared by one or more households and arranged in a circular or semi-circular enclosure around a common yard where social activities take place, compounds dominate the rural landscape, with sleeping areas, kitchens, and toilets existing as independent structures. The rare constructions with more than one sleeping room indicate owners of greater financial means than those made up of sun-dried mud bricks.

Different ethnic groups live in the region, sharing a vernacular language, Jula, and a variety of social habits and norms that allow for exchange, alliances and coexistence (Galvan 2006). These commonalities led to the employment of similar concepts throughout the study communities and the possibility, for the purpose of this study, of referring to similar lifeways. Most individuals identify as Toussian in Toma, Karaboro in Niakore, and Turka in Ouloumeni—ethnic groups that currently practice patrilineality and patrilocality, although historically are of mixed heritage (Koné 2002). A ‘family’ (*sómɔgɔw*, lit. ‘persons of the house’) is recognised through its male head of household and operates under his authority; children belong to his side of the parentage, and he decides upon property, including bednets. Polygyny is legal and discrete from religious beliefs. In rural areas, participants associated polygyny with the ownership of farming fields and with the possibility of providing each wife and their children with a kitchen and lodging space. In Toma, characterised by the lack of arable land, polygynous households were relatively scarce (five out of 22 participant households) in comparison with Niakore (nine out of 16) and Ouloumeni (eight out of nine). Participants broadly considered four children per wife (not per husband) as a ‘good-sized family’, although some worried about the increasing scarcity of resources.

Women access independent income only with their husbands’ permission—a circumstance that reflects proportionally on women’s decision-making power (Richards et al. 2013). Gender norms and age cohorts refract through constructions of time and space, determining who occupies specific areas (the sharing of a room, moving to an independent housing structure), partakes of a meal (commonly, with individuals of the same gender), and is in charge of making decisions.

Toma, Niakore, and Ouloumeni connect to the municipal water distribution (wells and pumps) and some private water sources (locked taps). All households lack electricity; few can afford solar panels. The diverse ecological circumstances of each village reflect the communities’ livelihoods, as well as their access to infrastructure and services (i.e. roads, health centres, schools).

### Toma

With its internal pathways made of sand and clay, Toma sits on one side of the paved artery that connects Banfora to the country’s main road network and a health centre. From October to June, children attend its local primary school, cycle daily to Banfora to attend middle school, or move in with relatives or into shared dormitories in one of the country’s main urban centres to pursue further education—a practice common in the region.

Following government-led land expropriation in the 1970s and consequent resettling (cf. Giles-Vernick et al. 2011), the population today has minimal access to farming fields, enclosed within privately-owned sugarcane plantations. The plantations, not the farming fields, are now a primary source of income for the men, who work there from November until April; the women who earn a living move between local markets to trade in local produce.

### Niakore

Niakore is on an unpaved road far from the panel signalling its boundaries and past one of the largest gold-mining settlements in the region. Annual gravelling is meant to mitigate the effects of the rains, which render the routes unviable and isolate the community from the neighbouring villages for periods ranging from a few hours to several days. There is a primary school; older children move elsewhere to pursue further education for the length of the academic year (October to June).

Niakore is over 20 km away from the nearest health centre—further than the accessibility criteria recommended by the Ministry of Health. Extended fields farmed to grow produce tolerant of the drier environment (millet, peanuts, sesame, and cotton) characterise the landscape. The size of the land permits a broader distance between compounds, and there is no market. The other opportunities for men to earn a living are seasonal work in factories close to Banfora and trade. Women work in their husbands’ fields, and some occasionally take up short-term work (2–3 days) through a local farming co-operative.

### Ouloumeni

From one of the main roads of the region, a sharp turn leads through a dense grove of baobabs and shea trees, before opening out to the village of Ouloumeni. The area is known for its sources of water, rice fields, and the abundance of land, which is often let to individuals from neighbouring villages, including Toma. A large health centre is easily reachable from anywhere in the village.

Ouloumeni hosts a more diverse community than the other two villages, including some ethnic Fula. Gender roles loosely structured farming: women oversee the rice plantations and care for small animals, mainly goats; men farm corn and beans, and care for the cattle. Intense migration to and from the Ivory Coast mark seasonal variations: all participant households are connected to the Burkinabè diaspora, living between the two countries, migrating to follow the rainy season. Here, too, children commute to attend secondary school, travelling daily or weekly if hosted by relatives.

## Findings

### Bednet procurement

Participants considered bednets a valuable commodity against the nuisance of mosquitoes, other insects and nocturnal animals, and a means of protection against malaria, consistently hanging them in sleeping areas even when visibly worn. Only four participants, all men above the age of 45, declared that they never slept under a net, preferring (and being able to afford) the use of small fans.

Nets are either obtained through formal distribution, acquired through kinship, or purchased. Ante-natal care services complement national distribution and provide mothers with an additional bednet every time they deliver a baby at a health centre or hospital. Shortages of bednets become especially visible during the rainy/peak transmission season after households have devoted most resources to ritual celebrations or the preparation of farming fields (discussed below). Students and farmers return to the village for the holidays and to help with farming activities. Some migrants carry a net; most do not. Government- subsidised nets remain in the original residence where they are shared, as per distribution structuring, used by at least one more person—a fellow student, a wife, young children. Mahmood, a 20-year-old student, had recently returned to spend the school holidays in Ouloumeni when he explained,

I share the net. I leave it at the dorm because I share it, and I have no right over it. What if I or the other guy bring it to the village, and it tears? No, no. We leave it at the dorm. That way, we are sure we find it once back.

(Interview, Ouloumeni, June 2018)

**Figure 1:**
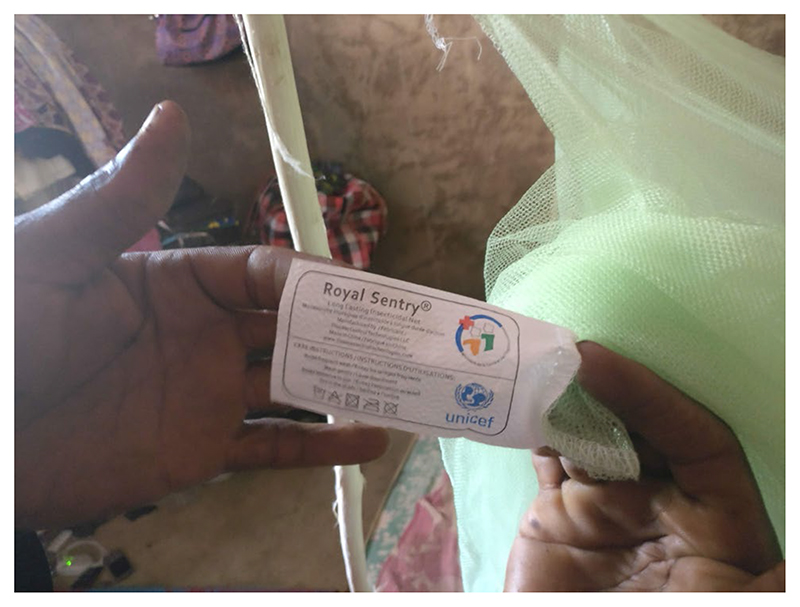
Bednets from the Ivory Coast could be easily identified by their green colour and the tag, bearing the symbol of the Ivorian Ministry of health.

Like other members of his compound, Mahmood had remained without a bednet until his 47-year-old father, Ahmed, returned from the Ivory Coast. Ahmed had brought several bednets, easily recognisable by their green colour and ministerial tag (Figure 1), to distribute to his relatives. Ahmed’s concerns were not unique, but his circumstances were fortunate. Other migrant farmers travelled domestically (i.e. with no access to different distribution strategies) and left their bednets behind with their families. The topic of bednet availability and allocation surfaced often. During a group conversation in Niakore, Tené—a young woman in a polygynous marriage—jokingly accused her husband of exposing her to malaria when sleeping together, as he preferred to use a fan. At the age of 25, Tené had given birth to her second child six months after the latest distribution campaign. After asking her husband to buy a new bednet, because the one she had was deteriorating, she confessed in private,

I have another. I keep it under the mattress. It’s still sealed! […] The children are small, but they grow up quickly. What then? What if mine tears? If I start asking now, by then maybe I’ll have convinced [my husband].

(Conversation, Niakore, November 2017)

Revealing a commonly shared apprehension over when (and if) the next distribution will take place, Tené misrepresented her bednet-to-user availability—what PMI (in the Introduction) accused the whole country of doing. However, Tené acted within the possibilities allowed by the precariousness of her circumstances: the youngest of four wives, she had no income, and a net’s market price corresponded to a daily seasonal wage (CFA 1500–2000, ~USD 2.5–3.3). The high frequency of informal procurement and the anxiety it triggered, such as in the cases illustrated above, demonstrate the value participants placed on nets and the inadequacy of the current post-positivist paradigm underpinning the distribution system in addressing the structural and social realities shaping the recipients’ needs and responses.

### Daily practices and nocturnal activities

Suleiman was a Muslim 78-year-old head of household in Ouloumeni. The head of a large household, upon agreeing to host the study, he remarked,

I sleep under a bednet every night—every night—but I still get malaria. Why is that? [chuckles] It must be the tea!

(Conversation, Ouloumeni, June 2018)

His comments reverberated throughout the study: participants consistently acknowledged the value of bednets but recognised that being outdoors in the evening and in the early morning—to work, rest, or socialise, as Suleiman indicated with his reference to drinking tea—meant that a bednet’s protection was limited.

Walking around the villages after dark, at any point throughout the year, we always found individuals engaging in different activities upon returning from the fields, from school, from the market. Socialising, receiving prominent guests, watching television (provided the solar panels were powerful enough), and sometimes sleeping, all take place in the open courtyards with exceptions mostly due to rainfall. Women prepare food in the courtyard or open kitchens, taking advantage of the cooler hours (early morning and late evening) to perform heavy tasks, such as fetching water and wood. The duty is shared with younger female members of the household as soon as they are old enough. Young boys partake in similar chores until the onset of adolescence, in line with a social construction of gender that intersects with age. These patterns were predictable and well-known to the research participants. Sibiri, a 56-year-old trader from Toma, promptly described the crowded courtyards during the hottest nights as soon as we mentioned sleep patterns as a study topic:

[In] March, April, this is what you see here [indicating the yard with a generous gesture of his hand]: everybody sleeping outdoors, even the women. [Under the tree] here, [nearby a house] there, even on chairs drinking tea or on the rug. It’s just too hot.

(Conversation, Toma, April 2018)

Sibiri was among the men who could afford a fan, so he and his family never slept outdoors. Outdoor presence varied with the season (Table 1), but participants identified this behaviour as ‘resting’ (rather than ‘sleeping’) until it was comfortable to be indoors. Sibiri’s younger brother and neighbour, Marc, highlighted this difference when asked where he slept:

[I sleep] indoors. […] But I go indoors late, around 23:00 or 00:00, when it is cooler. [My wife sleeps] indoors. Outdoors is not safe for a woman. And by the time she finishes working [outdoors], it is already cooler indoors.

(Interview, Toma, June 2017)

Perceived as more vulnerable, women and children are not allowed to spend longer outdoors than their head of household, who will wake up any family members resting outside to ensure that they go indoors before him. Several participants feared sleeping outdoors due to the risk of being attacked by thieves. However, in Toma, eight participants set up their net over a mattress outdoors throughout the dry season. These were men, widows, and older divorcees—i.e. individuals with a higher degree of self-determination and autonomy over available resources. Those who slept outdoors occasionally or only for a few hours did so on a plastic, braided mat covered with a piece of fabric, or on a reclined chair. The rough, external sides of the mat often damage the bednet, reducing its lifespan.

### Sleeping arrangements

Sleeping arrangements aggregate age, gender, and social status. Children below the age of five spend the longest time under a bednet: mothers are entirely responsible for their care and bring them to sleep indoors by 21:00–21:30 at the latest, with rare exceptions due to excessive heat. Children share the bed until pre-pubescence and are subsequently assigned to distinct areas—and bednets. Speaking about the fostered children for whom she cared, Fanta, Marc’s wife, noted,

[Next year] we will have to build a separate house. Amadou and Yasmina are no longer children, and they will soon become curious [of each other]. I told her to pay attention, even if she isn’t there yet [has not yet had her period], but they cannot stay together anymore.

(Conversation, Toma, July 2018)

As children age, sleeping arrangements adjust. Children move to sleep with other children from the compound; occasionally with their grandmothers. Thus, the number of bednets available one year might not suffice the next: changes in social roles brought by age negatively affect the time children spend under the protection of a bednet. Socio-economic status also plays a role: children from wealthier families rarely participate in domestic chores; fostered children and those from poorer households proportionally engage more in work supporting the family, spending longer outdoors in the early morning and evening. Gender dynamics affect activities and the use of space: children engage with the adults’ work and leisure, reconfirming physical and social separation between genders and situationally replacing age as the primary normative determinant (cf. Beverly and Whittemore 1993). Similarly, eating practices discourage the sharing of a meal between men and women, with women often eating with the youngest children, from different bowls and at different times.

Hinted at by Tenés teasing of her husband, a husband’s choice of bednet use constraints his wives’ agency. Couples do not necessarily share a bed—they may share it with the children of the same sex—although, in polygynous households, co-sleeping is more frequent. Wives take turns sharing the husband’s sleep space and, as such, the husband’s bednet preferences directly affect the wives’ protection. Separate sleeping arrangements are commonplace for women menstruating or experiencing post-partum bleeding, routinely altering their ability to comply with bednet use guidelines.

### Religion

Religious practices result in daily and seasonal exposure. In Muslim households, women exited their houses between 04:00 and 04:30 to warm the water for the ablutions of their husbands, children, and visitors in advance of Fajr, the first of five daily prayers. Animist celebrations follow an annual cycle—weddings between December and February; funerals, dedicated to the ritual celebration of the elderly (those above 50 years old) who passed away during the previous year, between March and May (Table 2). Organised with several months’ notice to allow extended family residing abroad to participate (which also requires the collection of the adequate funds), these weekend-long ceremonies involve tens if not hundreds of visitors, attending functions outdoors throughout the night.

One such instance was when, on the last weekend of April 2017, Johani’s family celebrated the memory of a relative in Bobo-Dioulasso, 85 km away from Toma. On a Friday morning, the men from the compound organised transport: borrowing small amounts of money from neighbours and friends, they ensured that the elderly could travel by bus and the youth by motorbike. Women and children, among which were co-wives Sara and Amina with a toddler and her 7-year-old sister, waited on the main road for less than an hour before hitchhiking a ride on a truck from well-meaning passers-by. Upon arrival, they were picked up by relatives who volunteered cars and bikes to bring them to the family compound, where hundreds of guests gathered.

A temporary kitchen accommodated a dozen women washing, peeling, cutting, and cooking the food. In the front yard, family members had arranged roughly a hundred plastic chairs, hired for the occasion for the more esteemed guests (the elderly or relatives occupying notable positions in society). Speeches inaugurated the celebration after dark, followed by a meal of rice, meat, fish, and vegetables; food remained available throughout the night. A group of drummers entertained the guests with music traditionally associated with the hosting family’s clan until midnight, when the dancing began in an adjacent larger area. The crowd stood in a large circle, at the centre of which individuals could perform to honour the deceased. Women served soft drinks, brewed coconut water (*bangi*) and millet (*dolo*) throughout, while Johani and his relatives (men or older women) challenged themselves to perform the most refined and daring choreographies. These exchanges reached an end only when unexpected rain poured from the sky, around 04:00. A partially covered space was available, but the communities shared the belief that the ritual drums interfere with the rain, strongly discouraging drumming during the rainy season.

Returning to the compound, Sara, Amina, and the other women organised a sleeping area by covering the floor of a repaired porch with mats. Shoes left at the entrance, this open space hosted over 30 women with their children, while the men slept in a neighbouring compound. Despite owning them, nobody brought bednets—the visit was short, and there would have been no way of hanging them—but their rest was brief: two hours later, the kitchen was buzzing with the preparation of millet and black tea for breakfast, and the courtyard full of guests waiting to be served before the second day of celebration began. Their exposure to mosquito bites, however, had been uninterrupted throughout the night.

### Labour

The communities’ vulnerability peaks during the rainy season, which is one of scarcity. Factory-based contracts come to an end in April, in-between the expenses incurred by the funerary ceremonies and the wait for newly purchased seeds, tools, and insecticides. The beginning of the rains, growing more unpredictable in recent years, is a source of apprehension. When scheduling an interview for the following day with Adama, a 38-year-old farmer from Niakore, he forewarned that he would not be available in case of rain. An unspoken rule in the communities, Adama kindly clarified:

We have two, three months [to farm]. What we do throughout these months, this is what we are going to live with for the rest of the year—there is no do-over, we cannot skip a day […]. This is what we eat.

(Conversation, Niakore, May 2018)

When it rains, the responsibility of farming to provide for one’s family takes precedence over any other activity: the farmers leave their homes before dawn and return after dark although these days coincide with a higher presence of mosquitoes. In Toma, Johani was working for the local sugarcane company when Yousef and Abdoulaye, two of his neighbours, joined him to share green tea one late May afternoon, and the conversation quickly veered towards the financial, physical, and social strain incurred with working for the company.

Factory workers attend a six-day, three-shift work rota (from 06:00 to 14:00; 14:00 to 22:00; 22:00 to 06:00) for a salary of CFA 28,000-42,000 (~$51-73) per month, focusing on the first phases of production: farming, product refinement and storage during the day; guarding the fields and the produce at night. While contracted, workers are routinely outside the protection of a bednet, and the company offers no alternative methods of protection against mosquito bites. Furthermore, the temporary nature of their employment limits access to factory-provided healthcare, for themselves and their immediate families. Yousef explained,

I am going to sign the contract in August. […] I will only start working…maybe… beginning of October. […] I sign the contract now because then I have [health] insurance, for me and my [nuclear] family. So, if we are sick—my wife or I, and the children—we can go to the [company] clinic, and they will treat us. For free. […] [This works] only when the contract is signed, not now.

(Group conversation, Toma, May 2018)

The companies find ways around providing such benefits by outsourcing employment to external contractors—incidentally, during the rainy season, when malaria transmission levels are higher. After the farming season had concluded, for instance, Johani was re-employed to patrol the fields:

Now I work the nights only, not the days. I guard the plantations so that people don’t come to steal anything. I go back and forth, every half-hour or so, from 18:30 to 05:30, then I come home. […]

We are not allowed to have bednets because they [the company management] say that, otherwise, we sleep. And the mosquitoes… Eh! They eat us alive! So, we use the bags… the rice bags, and keep our legs in them!

(Conversation, Toma, August 2018)

Trading offers a similar scenario. In Toma, the women leave the village before 05:30 to walk roughly 4 km to procure goods and set up small stalls on the main road by 07:30 at the latest. One such woman was Sara, whose husband had fled abroad to escape creditors, leaving her and her co-wife Amina to care for their seven children.

I reach [a nearby village], get the mangoes, then come back. [If I go] any later, it becomes too hot [to carry the weight]. Also, I miss some of the early buses that pass by on their way to Banfora.

(Interview, Toma, May 2018)

Sara explained that daily earnings vary considerably depending on the availability of crops: between 2017 and 2018, a kilo of corn could sell for CFA 200–300, tomatoes for CFA 800, and mangoes for as little as CFA 500 for roughly five kilos during high production season (April-May). Responsible for their children, these women leave their nets behind in the morning at the peak of Anopheline mosquito feeding hours in the region (Sanou et al. 2019).

**Figure 2:**
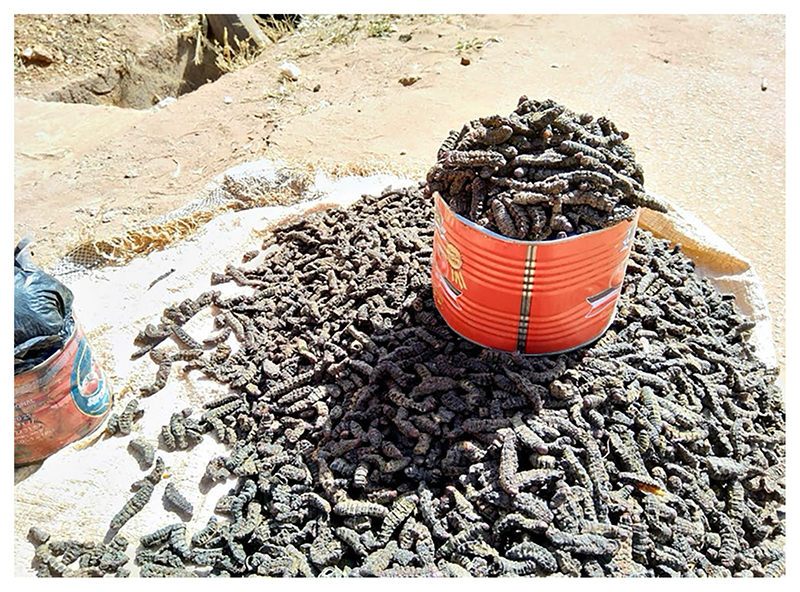
A tin of *caterpillars (Cirina butyrospermi)* sold at the market in Bobo-Dioulasso. The quantity of produce is measured through tins (versus weight) or units (when produce is rare).

As an increase in farming corresponds to food scarcity (FEWS 2018), shea trees *(Vitellaria paradoxa),* common in Toma and Ouloumeni, become instrumental to women’s earnings. In May, the trees produce nuts from which women make shea butter. More importantly, between July and August, the trees attract edible caterpillars *(Cirina butyrospermi)* that women and children harvest during the night to consume for months, fresh or desiccated, and sell in markets (Figure 2). Up to seven times more valuable than corn, caterpillars are financially fruitful, appealing, and represent an essential source of fat and protein. However, the harvesting takes place between 02:00 and 04:30—amid Anopheline feeding hours.

## Discussion

This paper has critically described how prospective bednet users live through and navigate structural poverty dynamics in which bednet use interweaves, moving the gaze from their expected technological primacy to their effectiveness within precarious assemblages. We showed that thinking of bednets as minimally intrusive devices is a way of misdirecting attention: the simplicity of the design and its mechanistic function make it so that bednets are fully integrated into the lives of the communities as ‘ordinary things in a network of transfers and delays’ (Stewart 2007, 21). Because of this integration, procurement and allocation reiterate gender- and age-based roles, in opposition to the flexibility of sleeping arrangements as imagined by the one-bednet-for-every-two-individuals ratio. Ordinary activities that include socialisation, labour, and religious practices permeate bednet use. Previous works identified specific factors, such as heat, comfort, and availability (Pulford et al. 2011) as ‘barriers’ to compliance. More recently, research extended to the circumstances that limit the beneficiaries’ ability to use bednets (Dunn, Le Mare, and Makungu 2016; Gryseels et al. 2015; Richards et al. 2013). Our study shows how bednet users enact compliance within agentive practices made available by their shifting structures.

Those who set themselves apart by systematically choosing not to use a bednet were rare, and participants valued the protection the device offered. The uncertainties and anxieties about procurement, while testifying to this perspective, lead to apparently contradictory behaviours: students and farmers—whose marginality is geographical, social, and financial (Agyepong and Manderson 1999)—prioritise long-term care of the net and their duties as heads of household; mothers negotiate the protection of their children to the detriment of the household’s shared resources. This affective dimension situationally moulds the form compliance takes: ensuring the protection of the vulnerable is a way to ‘comply’ with the ultimate aim of the intervention. Acts of proactive agency, these forms of compliance assemble affects, materiality, and decision-making.

Even when bednets were available, Feachem et al.’s argument (see Introduction) continues to face the issue of limited use. Bednets delegate and impose a specific behaviour on the beneficiaries. Like the clients in Brodwin’s (2010) work on the medication cassette, and echoing the ideological nature of compliance highlighted by Trostle’s (1988) analysis, the communities become responsible for ‘correct’ bednet use the moment they receive them, as nets channel expectations that reflect technological, political, and moral modes of ‘ordering’ the social. The cap on the number of bednets available to each household, for instance, presupposes that individuals will sleep (at least) in pairs, neglecting the generative role of age, gender, cultural norms, and migratory flows. Similarly, the assumption that sleeping under a net implies protection throughout Anopheline mosquito feeding hours (18:00–06:00) is fiction, informally recognised by the research community and explicitly acknowledged by the users. Adherence to bednet use is less mechanistic than the pharmacological compliance required by the medication cassette, but the framework used to assess compliance does not adapt. The numerical primacy that guides this approach is self-validating, further used to assess the efficacy of the intervention in what Tichenor (2017, 7) defines as ‘practices of certainty making.’ As such, numerical indicators record the uneven process that is compliance by exclusion, as ‘data left out’ (Tichenor 2017, 4). These data speak for micro- and macro-politics. Once delimited, the fluidity of these circumstances is lost and, alone, they remain insufficient to explain how compliance is enacted.

Participants complied with bednet use. Standardised assessments proved inadequate to capture the enactment of their compliance and, consequently, standardised interventions to improve it. By framing compliance as assemblage, we de-structure and decentre the phenomenon as a bounded sum of ‘factors’ and shift our focus from ‘determinants of compliance’ to ‘compliance as a process’ in relation to structural forces external to it yet constitutive of its performance. Responding to Trostle’s (1988) call for a re-conceptualisation of compliance, we turned the question from ‘if’ to ‘how’ communities use bednets, freeing the issue from an inadequate framework and support the analysis of its actualisation.

## Conclusions

This study contextualised bednet use within the structural circumstances in which it occurs, inviting a rethinking of the way we assess and evaluate ‘use’ and ‘compliance’ within the implementation of integrated vector-control management. The numerical indicators used to measure compliance do not address the discrepancies that entangle social identities, ecological factors, and the regularities of everyday life. Rather than asking whether individuals sleep under a bednet, we focused on the activities that bind bednet use to mundane realities as fundamental aspects of its effectiveness. Maps of human activities should be integrated into the planning of interventions. Discussing seasons as merely wet or dry is insufficient to address seasonal human activities, which are predictably more varied than those of mosquitoes. By framing ‘compliance’ in terms of assemblage, we do not offer a solution—one would, by definition, be inadequate—but a lateral perspective that positions complexities as quintessential to the existence of compliance itself.

## Figures and Tables

**Table 1:** Time individuals spend indoors (coloured) and outdoors (in white). Table 1a refers to the dry and hot season (March-May); Table 1b to the rainy (June-Sept) and cooler (Nov-Jan) seasons. Different patterns can be noticed in the indoor-outdoor movement of children (green), women (mustard), and men (brown). Lighter shades are used to highlight variations based on age and activities.

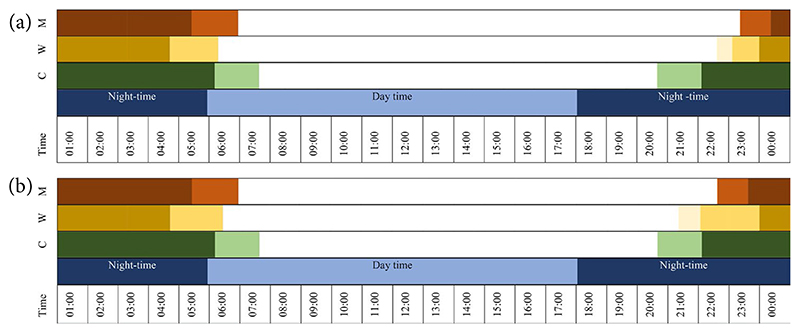

**Table 2:** Annual activities common to all communities in the region with reference to temperature, religious rites and ceremonies, farming, paid labour, and school year.